# Phylogenomic analysis uncovers the evolutionary history of nutrition and infection mode in rice blast fungus and other Magnaporthales

**DOI:** 10.1038/srep09448

**Published:** 2015-03-30

**Authors:** Jing Luo, Huan Qiu, Guohong Cai, Nicole E. Wagner, Debashish Bhattacharya, Ning Zhang

**Affiliations:** 1 Department of Plant Biology and Pathology, Rutgers University, Foran Hall 201, 59 Dudley Road, New Brunswick, New Jersey 08901; 2 Department of Ecology, Evolution, and Natural Resources, Rutgers University, Foran Hall 102, 59 Dudley Road, New Brunswick, New Jersey 08901; 3 National Animal Disease Center, USDA, PO Box 70, 1920 Dayton Ave, Ames, Iowa 50010; 4 Department of Biochemistry and Microbiology, Rutgers University, 76 Lipman Drive, New Brunswick, New Jersey 08901

## Abstract

The order Magnaporthales (Ascomycota, Fungi) includes devastating pathogens of cereals, such as the rice blast fungus *Pyricularia* (*Magnaporthe*) *oryzae*, which is a model in host-pathogen interaction studies. Magnaporthales also includes saprotrophic species associated with grass roots and submerged wood. Despite its scientific and economic importance, the phylogenetic position of Magnaporthales within Sordariomycetes and the interrelationships of its constituent taxa, remain controversial. In this study, we generated novel transcriptome data from 21 taxa that represent key Magnaporthales lineages of different infection and nutrition modes and phenotypes. Phylogenomic analysis of >200 conserved genes allowed the reconstruction of a robust Sordariomycetes tree of life that placed the monophyletic group of Magnaporthales sister to Ophiostomatales. Among Magnaporthales, three major clades were recognized: 1) an early diverging clade A comprised of saprotrophs associated with submerged woods; 2) clade B that includes the rice blast fungus and other pathogens that cause blast diseases of monocot plants. These species infect the above-ground tissues of host plants using the penetration structure, appressorium; and 3) clade C comprised primarily of root-associated species that penetrate the root tissue with hyphopodia. The well-supported phylogenies provide a robust framework for elucidating evolution of pathogenesis, nutrition modes, and phenotypic characters in Magnaporthales.

Magnaporthales is an order in Ascomycota within Fungi that contains over 200 species, including pathogens of cereals and grasses as well as saprobes on submerged wood^1^,^2^,^3^. The best-studied species in Magnaporthales is the rice blast fungus *Pyricularia* *oryzae* (= *Magnaporthe oryzae*), which is one of the most devastating threats to food security worldwide^4^,^5^. This pathogen is better known for its *Pyricularia* asexual state (fig. 1), which has been used for decades as a paradigm for fungal leaf infection studies^6^,^7^. The rice blast fungus also has the recently discovered root-infecting capability^8^. *Gaeumannomyces graminis* (take-all pathogen) and *Magnaporthiopsis poae* (summer patch pathogen), two other model species in Magnaporthales, are root-infecting pathogens of cereals and grasses. In addition to the plant pathogens, several saprotrophic wood-inhabiting genera (e.g., *Pseudohalonectria* and *Ophioceras*) are also placed in Magnaporthales^9^.

The studies of Magnaporthales date back to the late 19th century when the rice stem rot fungus *Nakataea oryzae* (= *Magnaporthe salvinii*) and the crabgrass pathogen *Pyricularia grisea* were first recorded^10^,^11^. Cannon^12^ established the family Magnaporthaceae to accommodate these grass-associated fungi centered on *Magnaporthe*. Historically, Magnaporthaceae was placed in various orders in Ascomycota, such as Diaporthales^13^, Phyllachorales, and Xylariales^14^, due to a lack of robust phylogenetic evidence and paucity of convincing phenotypic characters. A new order Magnaporthales was recently proposed for these fungi^2^. Molecular phylogenetic studies indicated that Magnaporthales belongs to the subclass Sordariomycetidae (Sordariomycetes, Ascomycota). However, consensus regarding additional phylogenetic affinities has not yet been reached. For example, in Zhang and Blackwell's^15^ 18S rDNA tree, Magnaporthales formed a sister clade with Ophiostomatales. Huhndorf et al.^16^ generated a 28S rDNA tree that suggested a close relationship of Magnaporthales with Sordariales, Chaetosphaeriales and Boliniales. In the 18S rDNA tree of Thongkantha et al.^2^, Magnaporthales formed a sister clade to Diaporthales and Ophiostomatales. In the same paper, however, the 28S rDNA tree grouped it with Chaetosphaeriales and Sordariaceae. Zhang et al.^17^ generated a four-gene phylogeny and found the rice blast fungus to be most closely related to Diaporthales; however, no other Magnaporthales taxa were included in the analysis. Poor taxon sampling due to a lack of genome data from Magnaporthales and phylogenetic heterogeneity among gene loci^18^,^19^ likely explain the conflicting tree topologies in the studies described above. Indeed, the current public availability of genome sequences from Magnaporthales is limited to the three model species: *Pyricularia oryzae*, *Magnaporthiopsis poae*, and *Gaeumannomyces graminis* var. *tritici*^7^(www.broadinstitute.org).

Therefore, despite their pathogenic diversity and economic importance, several key questions regarding the evolution of Magnaporthales remain unanswered. 1) Does Magnaporthales constitute a monophyletic group? 2) What is the phylogenetic position of Magnaporthales within Sordariomycetes? 3) What are the phylogenetic relationships among key taxa in Magnaporthales? and, 4) How have pathogenicity and phenotypes evolved among Magnaporthales taxa? In order to address these issues, we reconstructed the Magnaporthales phylogeny using a phylogenomic approach. Phylogenomics can often overcome problems associated with single locus or multi-locus phylogenetic analyses. To this end, we generated transcriptome data (~420 M paired-end reads) from 21 species of Magnaporthales, of which five were chosen for draft genome sequencing. Our work resulted in the largest collection of genome data to date for this poorly studied group and offers the opportunity to resolve the Magnaporthales phylogeny and to understand the evolution of its constituent lineages.

## Results and Discussion

### Transcriptome sequencing from 21 Magnaporthales species

We generated ~420 M paired-end reads (totaling ~65 Gbp) from the 21 targeted Magnaporthales taxa (tables S1 and S2). These data were assembled into a total of 315 K contigs with 13–31 K contigs from each taxon (N50 = 1.3–2.1 Kbp). To aid detection of potential gene duplications among Magnaporthales (see Methods), five of these taxa were subjected to complete genome sequencing, resulting in 35–43 Mbp genome assemblies that encoded ~12 K genes from each lineage (table S3). The mRNA-seq data have been submitted to NCBI (SRP050514, Bioproject number: PRJNA269089), whereas the results of the genome sequences and assembled contigs are presented at the project (dblab.rutgers.edu/FunGI/). In this paper, we focused on reconstruction of the Magnaporthales tree of life based on a set of highly conserved “core” genes. To this end, we selected 309 individual CEGMA (Core Eukaryotic Genes Mapping Approach; Parra et al.^38^) gene markers that are largely free of gene duplication in Magnaporthales and other Ascomycota (see Methods).

### Phylogenomics resolves the Magnaporthales tree of life

**Figure 1:**
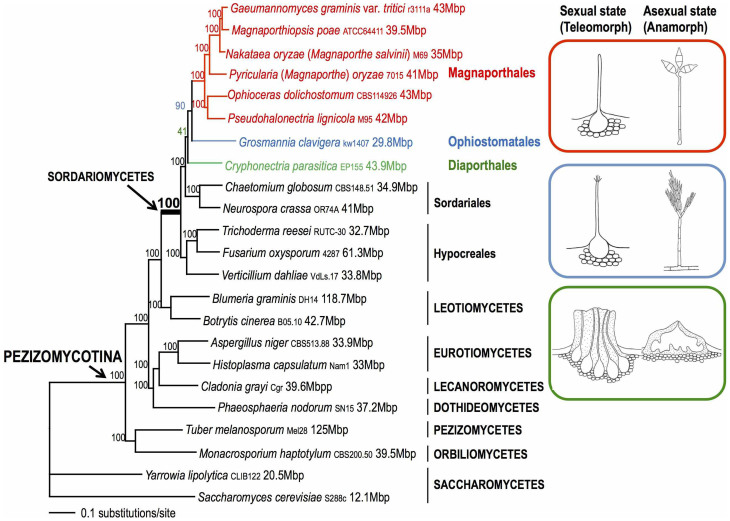
Maximum likelihood tree of 21 Pezizomycotina species and two Saccharomycetes used as outgroup species based on 83,616 amino acid positions derived from 226 genes. The morphology of the sexual and asexual states for Magnaporthales and closely related species is shown (right panel). The supporting values for each node were estimated using 1000 bootstrap replicates. The strain number and genome size for each species are provided. The schematic drawings were prepared by co-author J. Luo.

To address the phylogenetic position of Magnaporthales among Sordariomycetes, a maximum likelihood (ML) tree was generated using 83,616 aligned amino acids from 226 single-copy genes within six Magnaporthales species and 15 non-Magnaporthales species representing the major lineages of Pezizomycotina with two Saccharomycetes as outgroup (fig. 1, table S4). The ML tree provided strong bootstrap support (90–100%) for all interior nodes in the tree except for the union of *Cryphonectria parasitica*, *Grosmannia clavigera*, and Magnaporthales (41%). Bayesian inference (BI) using the same dataset showed a congruent and highly supported topology (see fig. S1). The topology of a well-supported coalescent model-based tree using this dataset of 23 species (fig. S2) was also consistent with the results shown in fig. 1, except for the swapping of branches containing *Cryphonectria parasitica* and the *Neurospora crassa*-*Chaetomium globosum* clade. Therefore, the phylogeny of the major Sordariomycetidae lineages appears to be largely resolved, except for the relationships among Sordariales, *Cryphonectria parasitica*, and *Grosmannia clavigera.*

**Figure 2:**
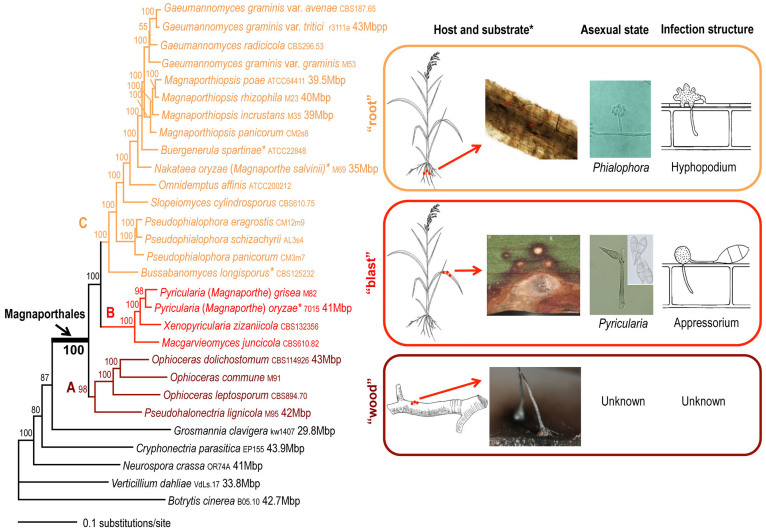
Maximum likelihood tree of 24 Magnaporthales species and five Sordariomycetes used as outgroup species based on 82,715 amino acid positions derived from 226 genes. The infection sites and the morphology of sexual and asexual states for the three major Magnaporthales clades are illustrated (right panel). The supporting values for each node were estimated using 1000 bootstrap replicates. The strain number and genome size (if available) for each species are provided. *Buergenerula spartinae, *Bussabanomyces longisporus*, *Nakataea oryzae*, and *Omnidemptus affinis* are associated with above-ground parts of host plants, which is an exception in clade C. *Pyricularia oryzae* is associated with both the leaf and root of the host plant, which is an exception in clade B. Drawings were done by author J. Luo. The photos were prepared by co-authors J. Luo and N. Zhang.

To study in detail the relationships within Magnaporthales, a ML tree was built using a 226-protein alignment that included 82,715 amino acids from 24 Magnaporthales and five outgroup taxa. This ML tree provided robust bootstrap support (98–100%) for the monophyly of the Magnaporthales and most interior nodes therein, except for the branch uniting *Gaeumannomyces radicicola* and *G. graminis* species (55%; fig. 2). The BI tree and the coalescent model-based tree using this dataset (fig. S3 and S4) resulted in very similar topologies. The minor differences between the multi-protein ML tree and the coalescent tree include: 1) The poorly supported paraphyletic relationship between *Gaeumannomyces graminis* var. *graminis* and *G. radicicola* in the ML tree (fig. 2), that was replaced with a monophyletic relationship in the coalescent tree (fig. S4); and 2) The positions of *Cryphonectria parasitica* and *Neurospora crassa* were swapped in the two trees.

In summary, we inferred well-supported phylogenies of Sordariomycetes and Magnaporthales using >200 conserved single-copy gene markers. Notably, the consistent topologies generated using multi-protein concatenation and the concatenation-free coalescent model suggests that our results are not biased by phylogenetic heterogeneity across genetic loci that may mislead multi-protein phylogeny construction (e.g., Song et al.^20^).

### The phylogenetic position of Magnaporthales within Sordariomycetes

Ascomycota, the largest group within Fungi includes three subphyla, Taphrinomycotina, Saccharomycotina, and Pezizomycotina, which are differentiated by the ascus dehiscence mechanism and fruiting body structure^21^,^22^. The order Magnaporthales, together with other filamentous ascomycetes, comprise the class Sordariomycetes that belongs to subphylum Pezizomycotina. Our phylogenomic analysis resolved relationships among the classes in Pezizomycotina included in the study (fig. 1). The Orbiliomycetes-Pezizomycetes clade (*Monacrosporium haptotylum* and *Tuber melanosporum*) represents an early diverging lineage in Pezizomycotina. The affinity between these two classes received stronger support from our analyses when compared to a previous study^21^. This result is consistent with shared Pezizomycotina characters such as the basic ascus type, the simple spore dehiscence mechanism, and the exposed hymenium^21^,^23^. The classes Eurotiomycetes (e.g., *Aspergillus niger*), Lecanoromycetes (*Cladonia grayi*), and Dothideomycetes (*Phaeosphaeria nodorum*) form a strongly supported clade. Their monophyletic relationship was recovered in earlier phylogenetic studies, however with lower bootstrap support than reported here^21^,^22^,^24^,^25^. Sordariomycetes and Leotiomycetes (e.g., *Blumeria graminis*) are the later diverging lineages and their monophyletic relationship is consistent with previous studies^18^,^19^,^21^,^24^. The shared characters between these two lineages include unitunicate and inoperculate asci. The latter is regarded as the advanced spore dispersal mechanism in Pezizomycotina^22^,^24^. Given these results, we surmise that single-copy CEGMA genes are useful phylogenetic markers that appear to recapitulate and consolidate previously suggested relationships amongst Pezizomycotina.

Although Magnaporthales formed a strongly supported monophyletic clade in Sordariomycetes (fig. 1), the phylogenetic position of this lineage relative to Ophiostomatales and Diaporthales is less well resolved using different phylogenetic methods (i.e., compare figs. 1, S1, S2). This was also the case in previous analyses of morphological and molecular data^2^,^16^,^21^. Nonetheless, our analyses support a sister group relationship between Magnaporthales and Ophiostomatales; the latter is represented here by the mountain pine beetle-associated blue stain fungus *Grosmannia clavigera*. This result is consistent with the shared characteristics between the two orders of non-stromatic perithecia and a hyphomycetous asexual state. In contrast, Diaporthales typically have stromatic perithecia and a coelomycetous asexual state (fig. 1). The grouping of Magnaporthales and Ophiostomatales (*Gromannia clavigera* or *Ophiostoma piliferum*) was also reported in two previous phylogenomic analyses that included fewer taxa^19^,^26^.

### Phylogenetic relationships among Magnaporthales taxa

In our phylogenomic analyses, three well-supported major clades are recognized within Magnaporthales (fig. 2). These three clades correspond to Magnaporthaceae, Pyriculariaceae and Ophioceraceae, the recently classified families in Magnaporthales that were identified using a 2-locus phylogeny^27^. Most species in clade C are associated with grass roots, use a hyphopodium as the penetration structure, and have a phialophora-like asexual state. The exceptions are *Buergenerula spartinae*, *Bussabanomyces longisporus*, *Nakataea oryzae*, and *Omnidemptus affinis* which are associated with the above-ground parts of host plants. The clade B species that cause blast diseases in Poaceae and other monocot plants use an appressorium as the host penetration structure and have a *Pyricularia* asexual state. The saprotrophic *Ophioceras* and *Pseudohalonectria* species form the basal clade A, which are associated with submerged wood tissue. Currently the asexual state of the majority of these species has not yet been observed^3^.

Recent phylogenetic studies support the idea that terrestrial habits and a saprotrophic lifestyle are plesiomorphies of the Sordariomycetes fungi^17^,^28^,^29^. Our results indicate that the clade A in Magnaporthales shifted from a terrestrial to an aquatic environment but maintained the ancestral saprotrophic lifestyle. In addition to the Wood clade in Magnaporthales, aquatic lineages occur in several other orders in Sordariomycetes, including Halosphaeriales, Sordariales, Diaporthales, and Xylariales^17^. Therefore, such a habit shift has apparently occurred several times in Sordariomycetes^17^,^28^.

Aerial and root infections are two strategies employed by plant pathogens to obtain host nutrients^30^. Species in clade B in Magnaporthales have adapted to aerial infection. The reproductive hyphae of the *Pyricularia* asexual state are long and erect, which facilitates conidium dispersal. After conidia attach to the host, a specialized structure known as an appressorium develops from conidia and penetrates the leaf cuticle using high turgor pressure, which is essential for fungal invasion of the host aerial parts (fig. 2)^30^. However, most clade C members appear to lack aerial infection ability and have adapted to root infection. Their asexual states are phialophora-like, which is characterized by simple and short reproductive hyphae^12^,^31^. These taxa form lobed and swollen hyphal structures named hyphopodia to penetrate the host root tissue and obtain nutrients (fig. 2)^30^.

Traditionally, ascospore morphology is the basis for genus and species delimitation in Magnaporthales and in many other ascomycetes. For example, in Magnaporthales, species that produce spindle-shaped ascospores are placed in *Magnaporthe*, whereas those that produce filiform ascospores are in *Gaeumannomyces*. The results of our study indicate that morphology-based generic concepts are unreliable as systematic markers; e.g., *Magnaporthe* and *Gaeumannomyces* are polyphyletic, whereas ecological and pathogenicity features correspond better to the phylogenetic history of Magnaporthales. The clear separation of the Blast clade and the *Magnaporthe* type species, *M. salvinii*, indicates that the rice blast fungus does not belong to the genus *Magnaporthe*, which was mistakenly classified in the 1970s based on ascospore morphology^14^. *Pyricularia*, the older asexual generic name for the rice blast fungus is informative and legitimate^32^, and therefore is suggested as the correct scientific name for this model taxon^27^,^33^,^34^. Taxonomic revision of *Magnaporthe*, *Gaeumannomyces*, and other Magnaporthales is underway or already published in other papers^35^,^36^.

In conclusion, our results demonstrate that Magnaporthales is a robustly supported monophyletic order within Sordariomycetes with its sister group provisionally being Ophiostomatales. The early diverging lineage of Magnaporthales (clade A) is mostly aquatic and saprotrophic on wood substrates. The pathogenicity of the remaining lineages is likely a derived feature with the B clade evolved to infect host aerial parts using appressoria and clade C adapted to plant root infection with hyphopodia. Our results significantly enrich sequence data from the order Magnaporthales and, more importantly, establish a framework for future comparative genomic and functional studies to address the evolution and ecology of this important lineage.

## Methods

### Fungal strains

Based on previously generated six-locus phylogenies^3^,^35^,^36^, we targeted 21 Magnaporthales species (table S1) for transcriptome sequencing, of which five (*Magnaporthe salvinii*, *Magnaporthiopsis incrustans*, *Magnaporthiopsis rhizophila*, *Ophioceras dolichostomum*, and *Pseudohalonectria lignicola*) were also used for whole-genome sequencing. The sequencing, annotation, and analyses of the genome data will be presented elsewhere (see table S3 for general information). The species sampled were obtained from the American Type Culture Collection, Manassas, Virginia (ATCC), the Centraalbureau voor Schimmelcultures Fungal Biodiversity Centre, the Netherlands (CBS) or the Rutgers Mycological Herbarium, New Brunswick, New Jersey (RUTPP).

### Transcriptome sequencing

The transcriptomes from 21 Magnaporthales taxa (table S1) were generated using Illumina sequencing. Total RNA was extracted using the RNeasy Plant Mini Kit (Qiagen, CA) from 1–2 week old mycelium grown in four different conditions: PDA under light, PDA in dark, minimal medium agar (MM; 10 g sucrose, 1 g Ca(NO_3_)_2_·4H_2_O, 0.2 g KH_2_PO_4_, 0.25 g MgSO_4_·7H_2_O, 0.15 g NaCl, 15 g agar in 1 liter distilled water) under light, and MM in dark. The TruSeq RNA Sample Preparation Kit (Illumina, CA) was used to construct mRNA libraries based on standard PCR methods. The libraries were sequenced using 300 cycle paired-end reagents on the MiSeq (Illumina) following the manufacturer's instructions.

### Transcriptome assembly and protein prediction

Transcriptome data from the five Magnaporthales taxa for which we generated whole-genome data (table S3) were trimmed to remove adaptors and assembled using Trinity with guidance of the corresponding in-house genome assemblies^37^. The mRNA-seq data from each of the 16 remaining Magnaporthales taxa (table S1 and S2) were cleaned and then assembled using CLC workbench 7 (http://www.clcbio.com/) under the default settings. Briefly, reads were cleaned to remove low-quality regions followed by adaptor sequence trimming. The resulting reads were used for *de novo* assembly. The reads were mapped to resulting contigs and the consensus contig sequences were optimized during the mapping procedure. The contig sequences were split at regions with <2X coverage and the consensus sequences (length ≥ 200 bp) were exported for further analyses.

Protein sequences from the five Magnaporthales species with whole-genome data were predicted using a combination of three gene prediction programs (i.e., SNAP, Augustus, and the self-training GeneMark-ES). The predicted protein sequences were used to build a local database that included complete proteomes from 20 other Ascomycota that are available in the public domain (table S4). The assembled contig sequences for the remaining 16 taxa were used as a query to search the local database using BLASTx (*e*-value ≤ 1e-10). The translated protein sequence from each query contig was derived from the BLASTx output.

### Selection of gene markers

We chose the CEGMA (Core Eukaryotic Genes Mapping Approach) gene set^38^ as markers to reconstruct the phylogeny. CEGMA proteins represent 458 KOGs (eukaryotic orthologous groups) that are conserved among eukaryotes^39^. To avoid misleading effects caused by gene duplication (e.g., Qiu et al.^40^), we limited the analyses to single-copy CEGMA genes. To this end, we downloaded the seven whole-proteome datasets that are annotated in the KOG database (http://www.ncbi.nlm.nih.gov/COG/), including those from nematode (*Caenorhabditis elegans*), common fruit fly (*Drosophila melanogaster*), human (*Homo sapiens*), mouse-ear cress (*Arabidopsis thaliana*), baker's yeast (*Saccharomyces cerevisiae*), fission yeast (*Schizosaccharomyces pombe*) and microsporidian parasite (*Encephalitozoon cuniculi*). We mapped a total of 25 complete proteomes, including those from the five in-house Magnaporthales taxa (table S3) and 20 other Ascomycota taxa (table S4), to these seven KOG-annotated proteomes based on the top hit from BLASTp (*e*-value ≤ 1e-5) searches. KOGs with more than one gene in two or more mapped Ascomycota taxa were removed, leaving 309 single-copy CEGMA KOG genes that were used for downstream analyses.

### Construction of single-protein alignments

We identified the genes corresponding to the 309 single-copy CEGMA genes in each of the tested taxa using a reciprocal-best-hit strategy. Ascomycota proteomes or proteins translated from EST contigs were used as query to search a local database comprising all CEGMA proteins using BLASTp (*e*-value ≤ 1e-5) and *vice versa*. For each CEGMA gene, sequences from different taxa were collected and aligned using MAFFT version 6.923b (--auto)^41^. The alignments were trimmed using Gblocks (-b4 = 5, -b5 = h)^42^. The resulting alignments were then processed with T-COFFEE version 9.03^43^ to remove poorly aligned residues (conservation score ≤ 5) within conserved sequence blocks. Columns with missing data (>80%) were removed and short alignments (<100 amino acids) were discarded. The remaining alignments were used for phylogenetic tree reconstruction. We generated a Pezizomycotina dataset (226 genes) including six Magnaporthales species and 15 other species representing major lineages in Pezizomycotina with two Saccharomycetes as outgroup (fig. 1), and a Magnaporthales-focused dataset (another 226 genes) including 24 Magnaporthales taxa and five closely related Sordariomycetes species (fig. 2).

### Phylogenetic reconstruction using concatenated multi-protein alignments

For each multi-protein alignment, we first inferred the best protein evolutionary model using Prottest version 3.2^44^. The LG + Γ + F model was identified as the top model for both multi-protein alignments (i.e., those used to infer the trees shown in figs. 1 and 2). Maximum likelihood trees were built using RAxML version 7.2.8^45^ under the selected model with branch support inferred using 1000 bootstrap replicates. Bayesian trees were built using MrBayes version 3.2.2^46^. Because MrBayes does not include LG model^47^, we modified the source code ‘model.c’ to incorporate it and then compiled the MPI-version executable. For each alignment, the analyses were performed with two independent runs each with one million generations. Trees were sampled every 100 generations. Consensus trees were built and the posterior probability values for all branches were calculated after removing 25% of the trees as burn-in (figs. S1 and S3).

### Tree reconstruction using the coalescent model

For the Pezizomycotina dataset, we reconstructed a maximum pseudo-likelihood tree (fig. S2) using a coalescent model that does not require the concatenation of multiple single-gene alignments^48^. Statistical support was estimated using 100 multilocus replicates generated using Seo's method^49^. For each replicate, we randomly sampled 226 genes with replacement. For each sampled gene, a pseudo-alignment was generated by random sampling of amino acid site from the corresponding original alignment with replacement. A ML tree was built for each pseudo-alignment using IQtree^50^ under the best amino acid evolutionary model selected on the fly. The ML trees were rooted using *Yarrowia lipolytica* or *Monacrosporium haptotylum* (in case of missing data in *Y. lipolytica*) as outgroup. The rooted trees were then used for maximum pseudo-likelihood tree construction using MP-EST^48^ under the default settings. The resulting 100 maximum pseudo-likelihood replicate trees were summarized following a majority rule using ‘Consense’ function in the Phylip package (http://evolution.genetics.washington.edu/phylip.html). The coalescent model-based tree for the Magnaporthales-focused dataset was generated following a similar procedure (fig. S4). The single gene trees were rooted using *Verticillium dahliae* or *Botrytis cinerea* (in case of missing data in *V. dahliae*).

## Author Contributions

J.L. and G.C. conducted most of the experiments in collaboration with N.E.W. who was in charge of Illumina DNA sequencing. H.Q. and G.C. completed data analysis. N.Z. and D.B. conceived and directed the project. J.L., H.Q., G.C., D.B. and N.Z. wrote and reviewed the manuscript.

## Supplementary Material

Supplementary Information Supplementary Information

## References

[b1] Besi M. I., Tucker S. L. & Sesma A. *Magnaporthe* and its relatives. In: Encyclopedia of Life Sciences (John Wiley & Sons, Chichester, 2009).

[b2] Thongkantha S. *et al.* Molecular phylogeny of Magnaporthaceae (Sordariomycetes) with a new species *Ophioceras chiangdaoense* from *Dracaena loureiroi* in Thailand. Fungal Divers. 34, 157–173 (2009).

[b3] Zhang N., Zhao S. & Shen Q. A six-gene phylogeny reveals the evolution of mode of infection in the rice blast fungus and allied species. Mycologia 103, 1267–1276 (2011). 21642347 10.3852/11-022

[b4] Ou S. H. Rice diseases (Kew, Surrey, UK: Commonwealth Mycological Institute, 1985).

[b5] Couch B. C. & Kohn L. M. A multilocus gene genealogy concordant with host preference indicates segregation of a new species, *Magnaporthe oryzae*, from *M. grisea*. Mycologia 94, 683–693 (2002). 21156541 10.1080/15572536.2003.11833196

[b6] Valent B. Rice blast as a model system for plant pathology. Phytopathology 80, 33–36 (1990).

[b7] Dean R. A. *et al.* The genome sequence of the rice blast fungus *Magnaporthe grisea*. Nature 434, 980–986 (2005). 15846337 10.1038/nature03449

[b8] Sesma A. & Osbourn A. E. The rice leaf blast pathogen undergoes developmental processes typical of root-infecting fungi. Nature 431, 582–586 (2004). 15457264 10.1038/nature02880

[b9] Shearer C. A. *Pseudohalonectria* (Lasiosphaeriaceae), an antagonistic genus from wood in freshwater. Can. J. Bot. 67, 1944–1955 (1989).

[b10] Cattaneo A. Sullo *Sclerotium* *oryzae*, nuovo parasita vegetale che ha devastato nel corrente anno molte risaje di Lombardia e del Novarese. Rendic. R. Lombard., Milano, 2 ser., 9, 801–807 (1876).

[b11] Saccardo P. A. Fungorum extra-europaeorum Pugillus. Michelia 2, 136–149 (1880).

[b12] Cannon P. F. The newly recognized family Magnaporthaceae and its interrelationships. Syst. Ascomycetum 13, 25–42 (1994).

[b13] Krause R. A. & Webster R. K. The morphology, taxonomy, and sexuality of the rice stem rot fungus, *Magnaporthe salvinii* (*Leptosphaeria salvinii*). Mycologia 64, 103–114 (1972).

[b14] Barr M. E. *Magnaporthe*, *Telimenella*, and *Hyponectria* (Physosporellaceae). Mycologia 69, 952–965 (1977).

[b15] Zhang N. & Blackwell M. Molecular phylogeny of dogwood anthracnose fungus (*Discula destructiva*) and the Diaporthales. Mycologia 93, 355–365 (2001).

[b16] Huhndorf S. M., Greif M., Mugambi G. K. & Miller A. N. Two new genera in the Magnaporthaceae, a new addition to *Ceratosphaeria* and two new species of *Lentomitella*. Mycologia 100, 940–955 (2008). 19202848 10.3852/08-037

[b17] Zhang N. *et al.* An overview of the systematics of the Sordariomycetes based on a four-gene phylogeny. Mycologia 98, 1076–1087 (2006). 17486982 10.3852/mycologia.98.6.1076

[b18] Fitzpatrick D. A., Logue M. E., Stajich J. E. & Butler G. A. fungal phylogeny based on 42 complete genomes derived from supertree and combined gene analysis. BMC Evol. Biol. 6, 99 (2006). 17121679 10.1186/1471-2148-6-99 PMC1679813

[b19] Ebersberger I. *et al.* A consistent phylogenetic backbone for the fungi. Mol. Biol. Evol. 29, 1319–1334 (2012). 22114356 10.1093/molbev/msr285 PMC3339314

[b20] Song S., Liu L., Edwards S. V. & Wu S. Resolving conflict in eutherian mammal phylogeny using phylogenomics and the multispecies coalescent model. Proc. Natl. Acad. Sci. USA 109, 14942–14947 (2012). 22930817 10.1073/pnas.1211733109 PMC3443116

[b21] Spatafora J. W. *et al.* A five-gene phylogeny of Pezizomycotina. Mycologia 98**,** 1018–1028 (2006). 17486977 10.3852/mycologia.98.6.1018

[b22] Zhuang W. & Liu C. What an rRNA secondary structure tells about phylogeny of fungi in Ascomycota with emphasis on evolution of major types of ascus. PLoS One 7, e47546 (2012). 23110078 10.1371/journal.pone.0047546 PMC3482189

[b23] Kumar T. K., Healy R., Spatafora J. W., Blackwell M. & McLaughlin D. J. *Orbilia* ultrastructure, character evolution and phylogeny of Pezizomycotina. Mycologia 104, 462–476 (2012). 22075787 10.3852/11-213

[b24] Robbertse B., Reeves J. B., Schoch C. L. & Spatafora J. W. A phylogenomic analysis of the Ascomycota. Fungal Genet. Biol. 43, 715–725 (2006). 16781175 10.1016/j.fgb.2006.05.001

[b25] Hibbett D. S. *et al.* A higher-level phylogenetic classification of the Fungi. Mycol Res. 111, 509–547 (2007). 17572334 10.1016/j.mycres.2007.03.004

[b26] DiGuistini S. *et al.* Genome and transcriptome analyses of the mountain pine beetle–fungal symbiont *Grosmannia clavigera*, a lodgepole pine pathogen. Proc. Natl. Acad. Sci. USA 108, 2504–2509 (2011). 21262841 10.1073/pnas.1011289108 PMC3038703

[b27] Klaubauf S. *et al.* Resolving the polyphyletic nature of *Pyricularia* (Pyriculariaceae). Stud. Mycol. 79, 85–120 (2014). 25492987 10.1016/j.simyco.2014.09.004 PMC4255532

[b28] Vijaykrishna D., Jeewon R. & Hyde K. D. Molecular taxonomy, origins and evolution of freshwater ascomycetes. Fungal Divers. 23, 351–390 (2006).

[b29] Schoch C. L. *et al.* The Ascomycota tree of life: a phylum-wide phylogeny clarifies the origin and evolution of fundamental reproductive and ecological traits. Syst. Biol. 58, 224–239 (2009). 20525580 10.1093/sysbio/syp020

[b30] Marcel S., Sawers R., Oakeley E., Angliker H. & Paszkowski U. Tissue-adapted invasion strategies of the rice blast fungus *Magnaporthe oryzae*. The Plant Cell 22, 3177–3187 (2010). 20858844 10.1105/tpc.110.078048 PMC2965542

[b31] Gams W. *Phialophora* and some similar morphologically little-differentiated anamorphs of divergent ascomycetes. Stud. Mycol. 45, 187–199 (2000).

[b32] McNeill J. *et al.* eds. International Code of Nomenclature for algae, fungi and plants (Melbourne Code). Adopted by the 18th International Botanical Congress, Melbourne, Australia, Jul 2011. (Regnum Veg, Koeltz Scientific Books, Germany, 2012).

[b33] Zhang N. *et al.* Impacts of the International Code of Nomenclature for algae, fungi and plants (Melbourne Code) on the scientific names of plant pathogenic fungi. Online. APSnet Feature. American Phytopathological Society, St. Paul, MN. (2013) (Date of access: 01/11/2014).

[b34] Tosa Y. & Chuma I. Classification and parasitic specialization of blast fungi. J. Gen. Plant Pathol. 80, 202–209 (2014).

[b35] Luo J. & Zhang N. *Magnaporthiopsis*, a new genus in Magnaporthaceae. Mycologia 105, 1019–1029 (2013). 23449077 10.3852/12-359

[b36] Luo J., Walsh E. & Zhang N. Four new species in Magnaporthaceae from grass roots in New Jersey Pine Barrens. Mycologia 106, 580–588 (2014). 24871590 10.3852/13-306

[b37] Grabherr M. G. *et al.* Full-length transcriptome assembly from RNA-Seq data without a reference genome. Nat. Biotech. 29, 644–652 (2011). 10.1038/nbt.1883 PMC3571712 21572440

[b38] Parra G., Bradnam K. & Korf I. CEGMA: a pipeline to accurately annotate core genes in eukaryotic genomes. Bioinformatics 23, 1061–1067 (2007). 17332020 10.1093/bioinformatics/btm071

[b39] Koonin E. V. *et al.* A comprehensive evolutionary classification of proteins encoded in complete eukaryotic genomes. Genome Biol. 5, R7 (2004). 14759257 10.1186/gb-2004-5-2-r7 PMC395751

[b40] Qiu H., Yang E. C., Bhattacharya D. & Yoon H. S. Ancient gene paralogy may mislead inference of plastid phylogeny. Mol. Biol. Evol. 29, 3333–3343 (2012). 22617952 10.1093/molbev/mss137

[b41] Katoh K. & Standley D. M. MAFFT: iterative refinement and additional methods. Methods Mol. Biol. 1079, 131–146 (2014). 24170399 10.1007/978-1-62703-646-7_8

[b42] Talavera G. & Castresana J. Improvement of phylogenies after removing divergent and ambiguously aligned blocks from protein sequence alignments. Syst Biol. 56, 564–577 (2007). 17654362 10.1080/10635150701472164

[b43] Notredame C., Higgins D. G. & Heringa J. T-Coffee: A novel method for fast and accurate multiple sequence alignment. J. Mol. Biol. 302, 205–217 (2000). 10964570 10.1006/jmbi.2000.4042

[b44] Darriba D., Taboada G. L., Doallo R. & Posada D. ProtTest 3: fast selection of best-fit models of protein evolution. Bioinformatics 27, 1164–1165 (2011). 21335321 10.1093/bioinformatics/btr088 PMC5215816

[b45] Stamatakis A. RAxML-VI-HPC: maximum likelihood-based phylogenetic analyses with thousands of taxa and mixed models. Bioinformatics 22, 2688–2690 (2006). 16928733 10.1093/bioinformatics/btl446

[b46] Ronquist F. *et al.* MrBayes 3.2: Efficient Bayesian phylogenetic inference and model choice across a large model space. Syst. Biol. 61, 539–542 (2012). 22357727 10.1093/sysbio/sys029 PMC3329765

[b47] Le S. Q. & Gascuel O. An improved general amino acid replacement matrix. Mol. Biol. Evol. 25, 1307–1320 (2008). 18367465 10.1093/molbev/msn067

[b48] Liu L., Yu L. & Edwards S. V. A maximum pseudo-likelihood approach for estimating species trees under the coalescent model. BMC Evol. Biol. 10, 302 (2010). 20937096 10.1186/1471-2148-10-302 PMC2976751

[b49] Seo T. K. Calculating bootstrap probabilities of phylogeny using multilocus sequence data. Mol. Biol. Evol. 25, 960–971 (2008). 18281270 10.1093/molbev/msn043

[b50] Minh B. Q., Nguyen M. A. & von Haeseler A. Ultrafast approximation for phylogenetic bootstrap. Mol. Biol. Evol. 30, 1188–1195 (2013). 23418397 10.1093/molbev/mst024 PMC3670741

