# Supplementary material for: Phylogenomic analysis uncovers the evolutionary history of nutrition and infection mode in rice blast fungus and other Magnaporthales
Source: Sci Rep. 2015 Mar 30;5:9448. doi: 10.1038/srep09448 (PMC4377577; doi:10.1038/srep09448)
Supplement: Supplementary Information [file srep09448-s1.pdf]

Supplemental information

Phylogenomic analysis uncovers the evolutionary history of nutrition and infection mode in rice blast fungus and other Magnaporthales

Jing Luo,[1 †] Huan Qiu,[2 †] Guohong Cai,[3] Debashish Bhattacharya,[2*] Ning Zhang[1,4*]

[1] Department of Plant Biology and Pathology, Rutgers University, Foran Hall 201, 59 Dudley Road, New Brunswick, New Jersey 08901

[2] Department of Ecology, Evolution, and Natural Resources, Rutgers University, Foran Hall 102, 59 Dudley Road, New Brunswick, New Jersey 08901

[3] National Animal Disease Center, USDA, PO Box 70, 1920 Dayton Ave, Ames, Iowa 50010

[4] Department of Biochemistry and Microbiology, Rutgers University, 76 Lipman Drive, New Brunswick, New Jersey 08901

[†] Equal contribution.

[*]Corresponding authors:

Debashish Bhattacharya, Email: debash.bhattacharya@gmail.com

Ning Zhang, Email: zhang@aesop.rutgers.edu

Table S1. The Magnaporthales taxa sampled in this study.

Taxa	Isolate no.	Source	Host
Buergenerula spartinae	ATCC22848	Rhode Island, USA	internode parts of *Spartina alterniflora*
Bussabanomyces longisporus	CBS125232	Chiang Mai, Thailand	leaves of *Amomum siamense*
Gaeumannomyces graminis var. *avenae*	CBS187.65	Netherlands	roots of *Avena sativa*
Gaeumannomyces graminis var. *graminis*	M53	Florida, USA	Poaceae sp.
Gaeumannomyces radicicola	CBS296.53	Ontario, Canada	roots of *Zea mays*
Macgarvieomyces juncicola	CBS610.82	Netherlands	stem base of *Juncus effusus*
Magnaporthiopsis incrustans *	M35	USA	Poaceae sp.
Magnaporthiopsis panicorum	CM2s8	New Jersey, USA	roots of *Panicum* sp.
Magnaporthiopsis rhizophila *	M23	USA	Poaceae sp.
Nakataea oryzae *	M69	Glenn county, California, USA	*Oryza* sp.
Omnidemptus affinis	ATCC200212	Queensland, Australia	leaves of *Panicum effusum* var. *effusum*
Ophioceras commune	M91	Yunnan, China	submerged wood
Ophioceras dolichostomum *	CBS114926	Hong Kong, China	submerged wood
Ophioceras leptosporum	CBS894.70	Exeter, UK	dead stems of dicot plant, probably *Urtica dioica*
Pseudohalonectria lignicola *	M95	Yunnan, China	submerged wood
Pseudophialophora eragrostis	CM12m9	New Jersey, USA	roots of *Eragrostis* sp.
Pseudophialophora panicorum	CM3m7	New Jersey, USA	roots of Poaceae sp.
Pseudophialophora schizachyrii	AL3s4	New Jersey, USA	roots of Poaceae sp.
Pyricularia grisea	M82	Tichnor, Arkansas, USA	*Digitaria* sp.
Slopeiomyces cylindrosporus	CBS610.75	UK	grass roots
Xenopyricularia zizaniicola	CBS132356	Kyoto, Japan	leaves of *Zizania latifolia*

* Taxa with whole-genome sequences

Table S2. Summary of Magnaporthales transcriptome data generated in this study.

Taxa	Isolate no.	Number of read-pairs (M)	Number of assembled contigs	N50	Total contig length
Buergenerula spartinae	ATCC22848	5.8	18,665	1,788	17,991,436
Bussabanomyces longisporus	CBS125232	7.1	18,882	1,848	19,415,458
Gaeumannomyces graminis var. *avenae*	CBS187.65	6.2	20,290	1,391	17,291,057
Gaeumannomyces graminis var. *graminis*	M53	6.4	18,822	2,003	20,086,722
Gaeumannomyces radicicola	CBS296.53	3.0	17,704	1,455	15,162,910
Macgarvieomyces juncicola	CBS610.82	5.4	13,133	2,143	16,638,136
Magnaporthiopsis incrustans *	M35	18.1	12,933	2,397	22,668,582
Magnaporthiopsis panicorum	CM2s8	6.8	22,259	1,341	18,633,977
Magnaporthiopsis rhizophila *	M23	28.7	12,210	2,396	21,748,038
Nakataea oryzae *	M69	16.5	12,077	2,148	19,552,960
Omnidemptus affinis	ATCC200212	7.2	18,489	1,911	19,563,674
Ophioceras commune	M91	5.3	20,608	1,671	19,184,729
Ophioceras dolichostomum *	CBS114926	32.6	12,519	2,666	24,039,271
Ophioceras leptosporum	CBS894.70	5.1	19,051	1,792	19,333,593
Pseudohalonectria lignicola *	M95	17.5	12,176	2,430	22,568,704
Pseudophialophora eragrostis	CM12m9	5.8	22,126	1,515	20,120,199
Pseudophialophora panicorum	CM3m7	5.6	20,384	1,733	19,908,431
Pseudophialophora schizachyrii	AL3s4	5.7	31,106	1,408	26,412,465
Pyricularia grisea	M82	7.4	15,380	2,061	18,169,371
Slopeiomyces cylindrosporus	CBS610.75	6.5	24,997	1,232	19,844,350
Xenopyricularia zizaniicola	CBS132356	5.3	13,885	1,915	14,974,402

* Taxa with whole-genome sequences. For these taxa, the statistics are based on annotated gene models using both genome assembly and transcriptome data.

Table S3. General information about five unpublished Magnaporthales whole-genome sequences used in this study.

**Taxa**	**Strain No.**	**No. of read-pairs (M)**	**Scaffold N50 (kb)**	**Contig N50 (kb)**	**Assembly Size (Mb)**	**# of Gene Models**
Magnaporthiopsis incrustans	M35	10.2	164.4	59.9	39	12933
Magnaporthiopsis rhizophila	M23	21.3	251.9	57.0	40	12210
Nakataea oryzae	M69	19.0	61.9	29.9	35	12077
Ophioceras dolichostomum	CBS114926	25.2	97.1	47.7	43	12519
Pseudohalonectria lignicola	M95	29.6	103.6	66.7	42	12176

Table S4. Proteome data from 20 Ascomycota taxa (including 3 Magnaporthales) that are available in the public domain.

Taxa	Strain No.	References	Data sources	Links
Aspergillus niger	CBS513.88	Pel et al. 2007	Ensembl Fungi release 22	http://fungi.ensembl.org/
Blumeria graminis	DH14	Spanu et al. 2010	DOE Joint Genome Institute	http://genome.jgi.doe.gov/Blugr1/Blugr1.home.html
Botrytis cinerea	B05.10	NA	Broad Institute of Harvard and MIT	http://www.broadinstitute.org/annotation/genome/botrytis_cinerea/MultiHome.html
Chaetomium globosum	CBS148.51	NA	Broad Institute of Harvard and MIT	http://www.broadinstitute.org/annotation/genome/chaetomium_globosum/Home.html
Cladonia grayi	Cgr	NA	DOE Joint Genome Institute	http://genome.jgi.doe.gov/Clagr2/Clagr2.home.html
Cryphonectria parasitica	EP155	NA	DOE Joint Genome Institute	http://genomeportal.jgi-psf.org/Crypa1/Crypa1.home.html
Fusarium oxysporum	4287	Ma et al. 2010	Ensembl Fungi release 22	http://fungi.ensembl.org/
Gaeumannomyces graminis var. *tritici**	r3111a	NA	Broad Institute of Harvard and MIT	Magnaporthe comparative Sequencing Project, Broad Institute of Harvard and MIT (http://www.broadinstitute.org/)
Grosmannia clavigera	kw1407	DiGuistini et al. 2009, 2011	DOE Joint Genome Institute	http://genome.jgi.doe.gov/Grocl1/Grocl1.home.html
Histoplasma capsulatum	Nam1	NA	Broad Institute of Harvard and MIT	http://www.broadinstitute.org/annotation/genome/histoplasma_capsulatum/MultiHome.html
*Magnaporthiopsis poae**	ATCC64411	NA	Broad Institute of Harvard and MIT	Magnaporthe comparative Sequencing Project, Broad Institute of Harvard and MIT (http://www.broadinstitute.org/)
Monacrosporium haptotylum	CBS200.50	Andersson et al. 2013, Meerupati et al. 2013	DOE Joint Genome Institute	http://genome.jgi.doe.gov/Monha1/Monha1.home.html
Neurospora crassa	OR74A	Galagan et al. 2003	Ensembl Fungi release 22	http://fungi.ensembl.org/
Phaeosphaeria nodorum	SN15	Hane et al. 2007, Bringans et al. 2009, Casey et al. 2010	Ensembl Fungi release 22	http://fungi.ensembl.org/
*Pyricularia oryzae**	7015	NA	Broad Institute of Harvard and MIT	Magnaporthe comparative Sequencing Project, Broad Institute of Harvard and MIT (http://www.broadinstitute.org/)
Saccharomyces cerevisiae	S288c	Cherry et al. 1997, Miura et al. 2006, Liti et al. 2009	Ensembl Fungi release 22	http://fungi.ensembl.org/
Trichoderma reesei	RUTC-30	Martinez et al. 2008	DOE Joint Genome Institute	http://genome.jgi-psf.org/Trire2/Trire2.home.html
Tuber melanosporum	Mel28	Martin et al. 2010	DOE Joint Genome Institute	http://genome.jgi.doe.gov/Tubme1/Tubme1.home.html
Verticillium dahliae	VdLs.17	NA	DOE Joint Genome Institute	http://genome.jgi.doe.gov/Verda1/Verda1.home.html
Yarrowia lipolytica	CLIB122	Dujon et al. 2004	Ensembl Fungi release 22	http://fungi.ensembl.org/

* Magnaporthales species

References:

Andersson KM, Meerupati T, Levander F, Friman E, Ahrén D, Tunlid A. 2013. Proteome of the nematode-trapping cells of the fungus *Monacrosporium haptotylum*. *Appl Environ Microbiol.* 79:4993–5004.

Bringans S, Hane JK, Casey T, Tan KC, Lipscombe R, Solomon PS, Oliver RP. 2009. Deep proteogenomics; high throughput gene validation by multidimensional liquid chromatography and mass spectrometry of proteins from the fungal wheat pathogen *Stagonospora nodorum*. *BMC Bioinformatics* 10:301.

Casey T, Solomon PS, Bringans S, Tan KC, Oliver RP, Lipscombe R. 2010. Quantitative proteomic analysis of G-protein signalling in *Stagonospora nodorum* using isobaric tags for relative and absolute quantification. *Proteomics* 10:38–47.

Cherry JM, Ball C, Weng S, Juvik G, Schmidt R, Adler C, Dunn B, Dwight S, Riles L, Mortimer RK, et al. 1997. Genetic and physical maps of *Saccharomyces cerevisiae*. *Nature* 387:67–73.

Diguistini S, Liao NY, Platt D, Robertson G, Seidel M, Chan SK, Docking TR, Birol I, Holt RA, Hirst M, et al. 2009. De novo genome sequence assembly of a filamentous fungus using Sanger, 454 and Illumina sequence data. *Genome Biol.* 10:R94.

DiGuistini S, Wang Y, Liao NY, Taylor G, Tanguay P, Feau N, Henrissat B, Chan SK, Hesse-Orce U, Alamouti SM, et al. 2011. Genome and transcriptome analyses of the mountain pine beetle-fungal symbiont *Grosmannia clavigera*, a lodgepole pine pathogen. *Proc Natl Acad Sci U S A.* 108:2504–2509.

Dujon B, Sherman D, Fischer G, Durrens P, Casaregola S, Lafontaine I, De Montigny J, Marck C, Neuvéglise C, Talla E, et al. 2004. Genome evolution in yeasts. *Nature* 430:35–44.

Galagan JE, Calvo SE, Borkovich KA, Selker EU, Read ND, Jaffe D, FitzHugh W, Ma LJ, Smirnov S, Purcell S,et al. 2003. The genome sequence of the filamentous fungus *Neurospora crassa*. *Nature* 422:859–868.

Hane JK, Lowe RG, Solomon PS, Tan KC, Schoch CL, Spatafora JW, Crous PW, Kodira C, Birren BW, Galagan JE, et al. 2007. Dothideomycete plant interactions illuminated by genome sequencing and EST analysis of the wheat pathogen *Stagonospora nodorum*. *Plant Cell* 19:3347–3368.

Liti G, Carter DM, Moses AM, Warringer J, Parts L, James SA, Davey RP, Roberts IN, Burt A, Koufopanou V, et al. 2009. Population genomics of domestic and wild yeasts. *Nature* 458:337–341.

Ma LJ, van der Does HC, Borkovich KA, Coleman JJ, Daboussi MJ, Di Pietro A, Dufresne M, Freitag M, Grabherr M, Henrissat B, et al. 2010. Comparative genomics reveals mobile pathogenicity chromosomes in *Fusarium*. *Nature* 464:367–373.

Martin F, Kohler A, Murat C, Balestrini R, Coutinho PM, Jaillon O, Montanini B, Morin E, Noel B, Percudani R, et al. 2010. Périgord black truffle genome uncovers evolutionary origins and mechanisms of symbiosis. *Nature* 464:1033–1038.

Martinez D, Berka RM, Henrissat B, Saloheimo M, Arvas M, Baker SE, Chapman J, Chertkov O, Coutinho PM, Cullen D, et al. 2008. Genome sequencing and analysis of the biomass-degrading fungus *Trichoderma reesei* (syn. *Hypocrea jecorina*). *Nat Biotechnol.* 26:553–560.

Meerupati T, Andersson KM, Friman E, Kumar D, Tunlid A, Ahrén D. 2013. Genomic mechanisms accounting for the adaptation to parasitism in nematode-trapping fungi. *PLoS Genet.* 9:e1003909.

Miura F, Kawaguchi N, Sese J, Toyoda A, Hattori M, Morishita S, Ito T. 2006. A large-scale full-length cDNA analysis to explore the budding yeast transcriptome. *Proc Natl Acad Sci U S A.* 103:17846–17851.

Pel HJ, de Winde JH, Archer DB, Dyer PS, Hofmann G, Schaap PJ, Turner G, de Vries RP, Albang R, Albermann K, et al. 2007. Genome sequencing and analysis of the versatile cell factory *Aspergillus niger* CBS 513.88. *Nat Biotechnol.* 25:221–231.

Spanu PD, Abbott JC, Amselem J, Burgis TA, Soanes DM, Stüber K, Ver Loren van Themaat E, Brown JK, Butcher SA, Gurr SJ, et al. 2010. Genome expansion and gene loss in powdery mildew fungi reveal tradeoffs in extreme parasitism. *Science* 330:1543–1546.

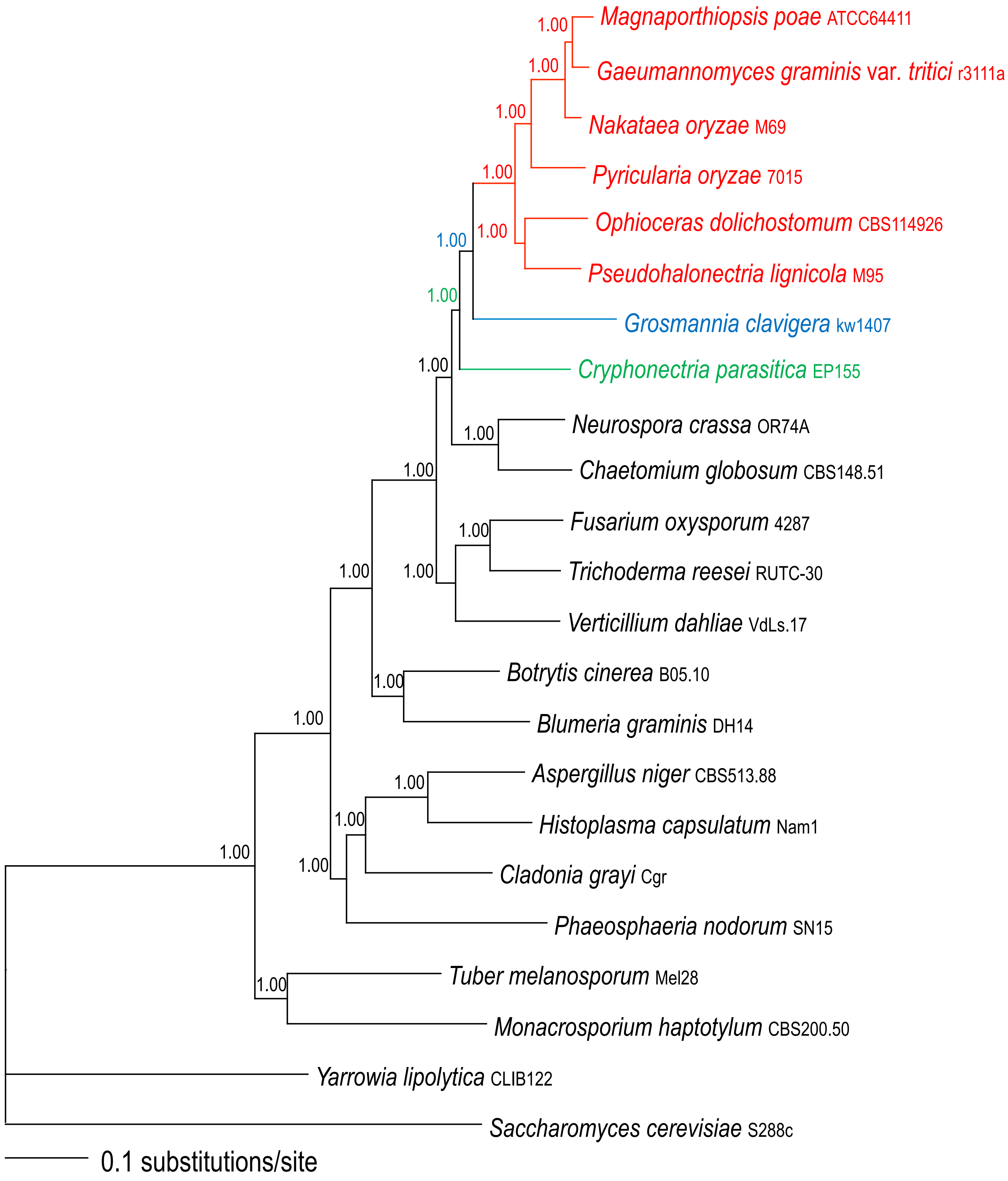

Fig. S1. Bayesian inference tree of 21 Pezizomycotina species and two Saccharomycetes used as outgroup species based on the LG + Γ + F model.

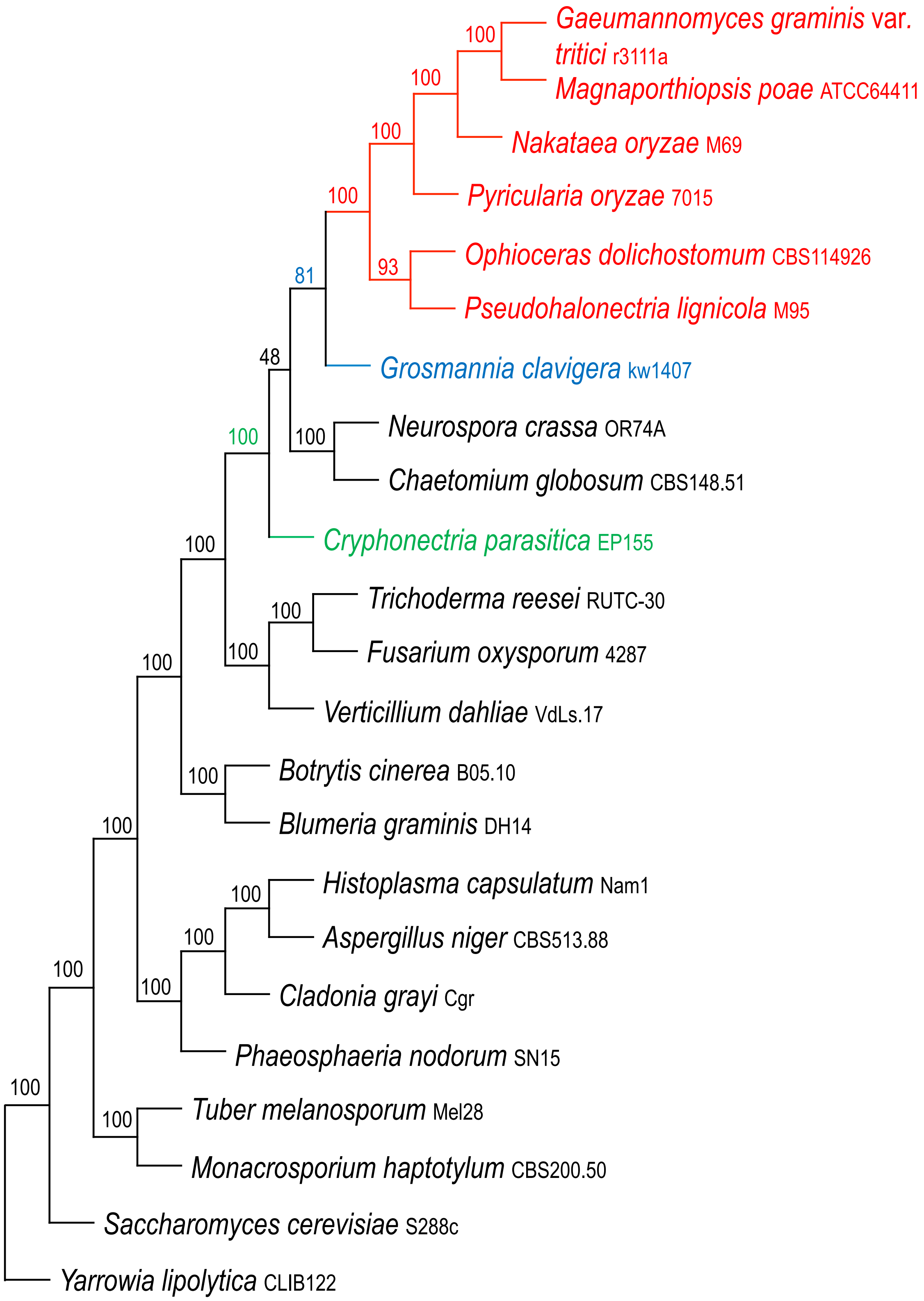

Fig. S2. Maximum pseudo-likelihood tree of 21 Pezizomycotina species and two Saccharomycetes used as outgroup species based on the coalescent model. The support values for each node were estimated using 100 multi-loci replicates. Note that the tree does not contain branch length

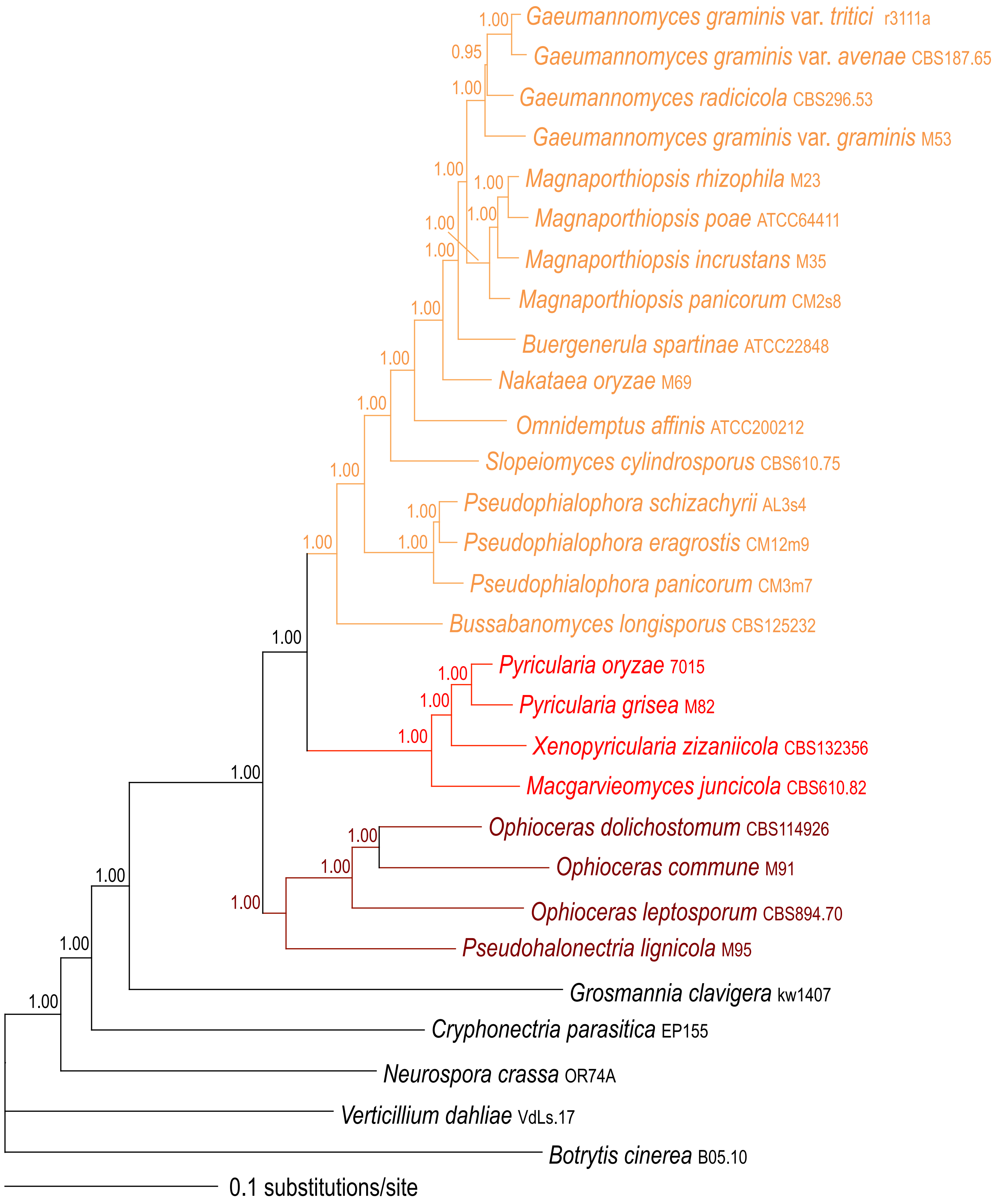

Fig. S3. Bayesian inference tree of 24 Magnaporthales species and five Sordariomycetes used as outgroup species based on the LG + Γ + F model.

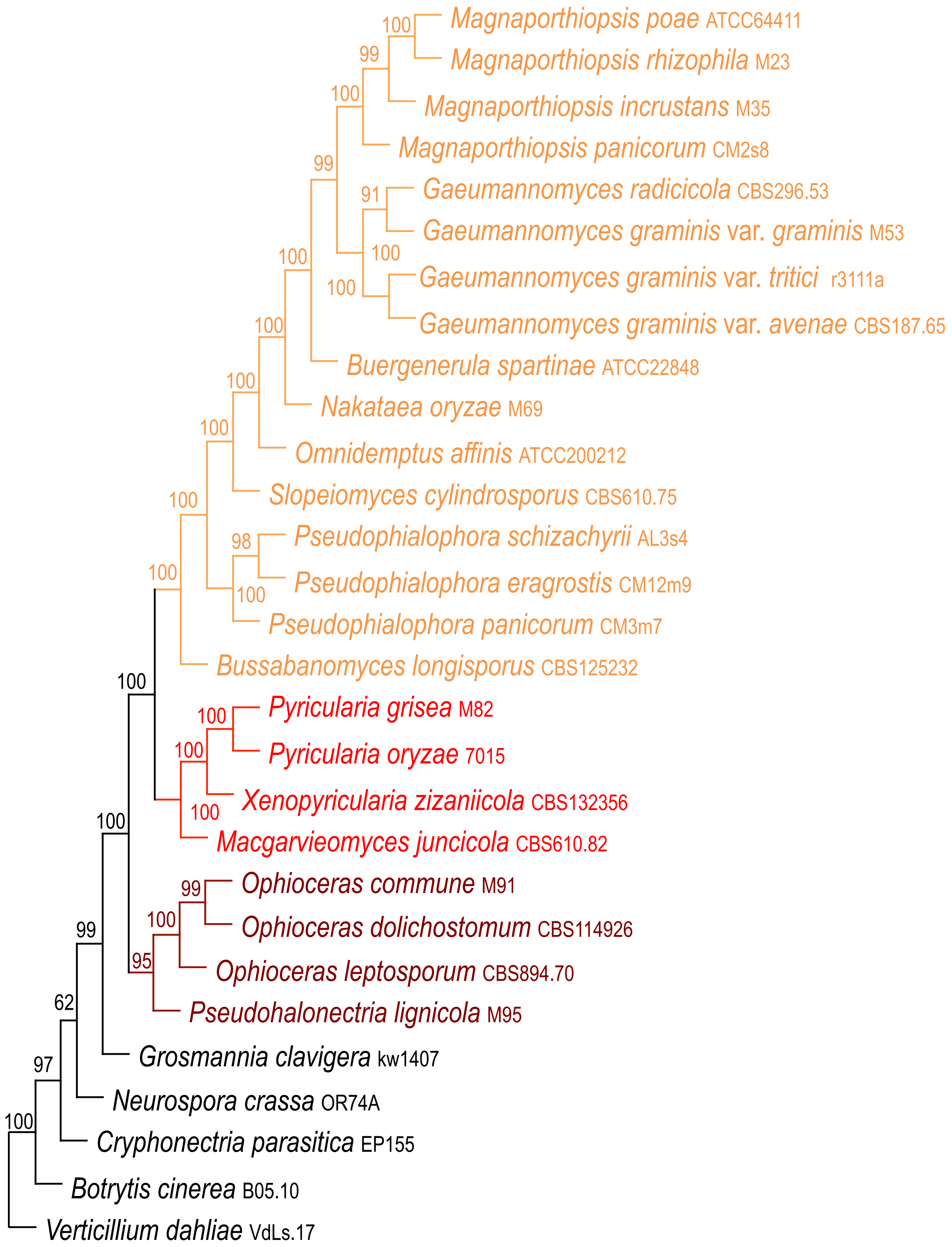

Fig. S4. Maximum pseudo-likelihood tree of 24 Magnaporthales species and five Sordariomycetes used as outgroup species based on the coalescent model. The supporting values for each node were estimated using 100 multi-loci replicates. Note that the tree does not contain branch length
